# Circulating complement factor H–related proteins 1 and 5 correlate with disease activity in IgA nephropathy

**DOI:** 10.1016/j.kint.2017.03.043

**Published:** 2017-10

**Authors:** Nicholas R. Medjeral-Thomas, Hannah J. Lomax-Browne, Hannah Beckwith, Michelle Willicombe, Adam G. McLean, Paul Brookes, Charles D. Pusey, Mario Falchi, H. Terence Cook, Matthew C. Pickering

**Affiliations:** 1 Centre for Complement and Inflammation Research, Imperial College London, UK; 2 Renal and Transplant Centre, Imperial College Healthcare NHS Trust, London, UK; 3 Histocompatibility & Immunogenetics, Imperial College Healthcare NHS Trust, London, UK; 4 Renal and Vascular Inflammation Section, Imperial College London, UK; 5 Department of Twin Research and Genetic Epidemiology, King's College London, UK

**Keywords:** complement, glomerular disease, IgA nephropathy

## Abstract

IgA nephropathy (IgAN) is a common cause of chronic kidney disease and end-stage renal failure, especially in young people. Due to a wide range of clinical outcomes and difficulty in predicting response to immunosuppression, we need to understand why and identify which patients with IgAN will develop progressive renal impairment. A deletion polymorphism affecting the genes encoding the complement factor H-related protein (FHR)-1 and FHR-3 is robustly associated with protection against IgAN. Some FHR proteins, including FHR-1 and FHR-5, antagonize the ability of complement factor H (fH), the major negative regulator of the complement alternative pathway, to inhibit complement activation on surfaces, a process termed fH deregulation. From a large cohort of patients, we demonstrated that plasma FHR-1 and the FHR-1/fH ratio were elevated in IgAN and associated with progressive disease. Plasma FHR-1 negatively correlated with eGFR but remained elevated in patients with IgAN with normal eGFR. Serum FHR5 was slightly elevated in IgAN but did not correlate with eGFR. Neither FHR5 levels nor the FHR-5/fH ratio was associated with progressive disease. However, higher serum FHR-5 levels were associated with a lack of response to immunosuppression, the presence of endocapillary hypercellularity, and histology scores of disease severity (the Oxford Classification MEST score). Thus, FHR-1 and FHR-5 have a role in IgAN disease progression.

IgA nephropathy (IgAN) is the most common primary glomerulopathy worldwide and is an important cause of chronic kidney disease, especially in young people.^1^ IgAN is associated with a wide range of clinical outcomes. Although 40% of patients will have reached end-stage renal disease within 20 years of diagnosis, 20% of patients will have preserved renal function with only minor urinary abnormalities.^2^ IgAN is characterized by dominant or codominant IgA-containing immune deposits on renal biopsy.^3^ The pathophysiology of IgAN associated with characteristic galactose-deficient IgA1 (gd-IgA1)-containing immune deposits is considered to be a 4-hit mechanism.^4^ However, the link between mesangial IgA deposition and the spectrum of clinical outcomes that are characteristic of IgAN is poorly understood. Therefore, we are limited in our ability to both identify patients for whom immunosuppression therapy is appropriate and develop novel therapeutic strategies. The current repertoire of clinical tools available to predict outcome and guide treatment strategies of IgAN, such as proteinuria or estimated glomerular filtration rate (eGFR), may reflect progressive glomerular scarring as well as active immunologically driven disease.

Systemic and renal complement activation in IgAN is well documented. However, the role of complement activation in IgAN pathogenesis remains poorly defined. Complement C3 accompanies IgA immunostaining in most diagnostic IgAN biopsies.^5^ Evidence of complement alternative pathway activation was identified in the plasma of 30% to 75% of IgAN adults^6^, ^7^ and correlated with proteinuria and the rate of renal function loss in a cohort of 50 IgAN patients.^7^ An association was demonstrated between mesangial C3 deposition, decreased serum C3 levels, and doubling of serum creatinine or reaching end-stage renal disease in a cohort of 343 IgAN patients.^8^ This demonstrated a link between histology and serum markers of complement activation in IgAN pathogenesis. Complement activation by the mannose-binding lectin (MBL) complement pathway in IgAN has also been demonstrated. MBL and MBL-associated serine protease (MASP)-1 were detected in 24% of renal biopsies from IgAN patients, but were identified in less than 3% of biopsies from patients with other forms of glomerulonephritis.^9^ Deposited MBL and MASP-1 were associated with C3b, C3c, and C5b9, which are markers of alternative and terminal complement pathway activity.^9^ Although this study did not show associations with markers of clinical outcomes, glomerular MBL and other MBL complement pathway components, including MASP-1/-3 and MASP-2, were found in 25% of a separate cohort of 60 IgAN patients, and these findings correlated with proteinuria and histologic features of severity.^10^

The ability of IgA to activate complement *in vitro*, predominantly through the alternative pathway, has also been demonstrated. IgA isolated from pooled human plasma triggers complement-dependent lysis of, and properdin deposition on, erythrocytes coated with mouse monoclonal antihuman IgA.^11^ Furthermore, a rat model of IgA-mediated glomerular inflammation demonstrated that polymeric but not monomeric IgA triggered mesangial C3 deposition and not C4 or C1q deposition.^12^

Recent genetic studies implicate a role for the complement factor H related (FHR) proteins in IgAN.^13^, ^14^, ^15^, ^16^ The FHR proteins may interfere with the regulatory functions of factor H (fH), the major negative regulator of complement C3 activation.^17^, ^18^ This process is referred to as fH deregulation.^19^, ^20^ The deletion polymorphism of the genes coding FHR-3 and FHR-1 (del*CFHR3-1*) is associated with protection from IgAN.^13^, ^14^, ^16^ A meta-analysis of approximately 20,000 individuals of different ethnicities estimated that the inheritance of the minor A allele at single-nucleotide polymorphism rs6677604, which tags the del*CFHR3-1* allele, reduced the risk of IgAN disease by 26% in heterozygosity and by 45% in homozygosity.^14^ Across populations worldwide, del*CFHR3-1* frequency exhibits marked differences in a pattern inverse to that of IgAN prevalence.^14^ An association has also been demonstrated between rs6677604 and histologic IgAN markers. In Chinese patients with IgAN, the rs6677604-A allele was associated with reduced mesangial C3 deposition, high serum fH levels, and low complement C3a levels but was not associated with clinical outcomes.^15^ Xie *et al.* showed an association of del*CFHR3-1* with reduced segmental sclerosis and tubular atrophy in IgAN.^16^ Although this was independent of eGFR and proteinuria, no other associations with clinical parameters or outcomes were demonstrated.

By enhancing complement activation in response to mesangial gd-IgA1-containing immune complexes, we hypothesized that fH deregulation influences disease severity in IgAN. In this study, we assessed circulating fH, FHR-1, and FHR-5 levels in IgAN patients who were stratified into cohorts with stable and progressive disease.

## Results

### Patient cohort

The patient cohort characteristics are summarized in Table 1. Using the criteria detailed in the Materials and Methods, 179 patients had progressive IgAN and 89 had stable IgAN. We had insufficient data to categorize 26 patients as having either progressive or stable IgAN. The cohort of patients meeting the criteria for progressive IgAN showed lower eGFR and higher systolic blood pressure than the stable IgAN cohort (Table 1). Consistent with previous reports,^21^, ^22^, ^23^ the median serum IgA and gd-IgA1 levels were higher in patients than in controls (Table 1). The progressive IgAN cohort showed lower median serum IgA and higher median serum gd-IgA1 levels than stable IgAN (Table 1). Similarly, of the patients who had received immunosuppression therapy, serum gd-IgA1 levels were higher in patients with progressive IgAN than stable IgAN after treatment (0.54 units [AU] vs. 0.44 AU, *P* = 0.04, Supplementary Figure S1A). Consistent with these data, we detected a negative correlation between serum gd-IgA1 levels and eGFRs at the sampling time point (Supplementary Figure S1B). The del*CFHR3-1* allele is associated with protection from IgAN.^13^ However, we did not observe any difference in *CFHR3* and *CFHR1* copy numbers between patients with stable IgAN and those with progressive IgAN (Table 1).

**Table 1:** Cohort characteristics

Variable	IgA nephropathy patients			Healthy controls (n = 161)
	Entire IgAN (n = 294)	Stable IgAN (n = 89)	Progressive IgAN (n = 179)	
Clinical features				
Male/Female	195/99	53/36	120/59	
Caucasian/Non-Caucasian	244/50	78/11	144/35	
Median age (range), yr	48.2 (18–84)	47.5 (18–82)	48.3 (19–84)	
Median eGFR, ml/min per 1.73 m^2^	52.7 (28.7–82.7)	74.8 (47.7–106)	45.8 (17.4–70.9)^a^	
Median urine PCR, mg/mmol	44 (16–117)	39 (13.5–89)	50 (17–144.5)	
Median antihypertensive drug classes per patient	1.6	1.4	1.6	
Patients with ACEi/ARB at enrolment, excluding dialysis patients, % (n)	73.7 (n = 199)	79.8 (n = 71)	77.3 (n = 109)	
Median systolic/diastolic blood pressure, mm Hg	134 (122–145)/79 (70–88) (n = 286)	128 (116–140)/77.5 (70–85) (n = 88)	136 (124–146)^b^/79 (70–88) (n = 174)	
Median follow-up duration, mo	55.0 (22.5–100.8)	71.4 (22.9–177.6)	50.7 (22.2–91.6)	
Reached ESRD, %	34.9 (n = 103)	0	57.5^c^ (n = 103)	
History of macroscopic hematuria, %	27.8 (n = 82)	44.9 (n = 40)	20.1^c^ (n = 36)	
Diagnosis of Henoch-Schonlein purpura, %	6.4 (n = 19)	0	8.9^c^ (n = 16)	
Laboratory measurements				
Median serum IgA, g/l	3.4 (2.9–4) (n = 293)	3.7 (3.2–4.3) (n = 88)	3.3 (2.7–3.8)^d^ (n = 178)	2.8 (2.2–3.2)^e^ (n = 57)
Median serum gd-IgA1, AU	0.50 (0.41–0.58) (n = 293)	0.47 (0.37–0.55) (n = 88)	0.52 (0.42–0.59)^f^ (n = 178)	0.42 (0.33–0.55)^g^ (n = 57)
*CFHR1*–2 copies/1 copy/no copies^h^				
n	183/101/9 (n = 293)	58/28/3	111/63/4 (n = 178)	85/45/3 (n = 133)
%	62.4/34.5/3.1	65.9/31.8/3.4	62.4/35.4/2.2	63.9/33.8/2.3
*CFHR3*–2 copies/1 copy/no copies				
n	185/100/8 (n = 293)	59/27/3	112/63/3 (n = 178)	
%	63.1/34.1/2.7	67/30.7/3.4	62.9/35.4/1.7	
Median plasma FHR-1, μg/ml	126.8 (86.4–158.6)	112.5 (74.7–149.3)	132.0 (88.6–162.8)	94.4 (70.5–119.6)^i^
Median plasma fH, μg/ml	153.8 (130.6–187.6)	155.1 (137.4–187.8)	150.1 (126.0–186.9)	152.5 (122.9–189.8)
Median plasma FHR-1:fH ratio	0.85 (0.55–1.10)	0.77 (0.46–0.98)	0.89 (0.59–1.16)^j^	0.68 (0.40–0.86)^k^
Median serum FHR-5, μg/ml	2.74 (2.07–3.64)	2.80 (2.07–3.49)	2.79 (2.08–4.03)	2.46 (1.79–3.67)^l^ (n = 158)

ACEi, angiotensin-converting enzyme inhibitor; ARB, angiotensin receptor blocker; AU, arbitrary units; CFHR, complement factor H-related; CI, confidence interval; ESRD, end-stage renal disease; fH, factor H; FHR-1/-5, factor H-related protein 1/5; gd-IgA1, galactose-deficient IgA1; PCR, protein-to-creatinine ratio; no, number.

Values within parentheses represent interquartile range and number analyzed if less than the respective cohort numbers.

a *P* < 0.0001 versus stable disease.

b *P* = 0.0014 versus stable disease.

c *P* < 0.0001 versus stable disease.

d *P* = 0.0007 versus stable disease.

e *P* < 0.0001 versus entire IgAN cohort.

f *P* = 0.01 versus stable disease.

g *P* = 0.015 versus entire IgAN cohort.

h 1 patient had 3 copies of *CFHR3* and was excluded from the analysis.

i *P* < 0.0001 versus entire IgAN cohort (difference between medians, 32.4 μg/ml; 95% CI, 19.9–37.6 μg/ml).

j *P* = 0.019 versus stable disease (difference between medians, 0.12; 95% CI, 0.02–0.2).

k *P* < 0.0001 versus entire IgAN cohort (difference between medians, 0.17; 95% CI, 0.12–0.24).

l *P* = 0.041 versus entire IgAN cohort (difference between medians, 0.28 μg/ml; 95% CI, 0.01–0.48 μg/ml).

### Plasma FHR-1 levels and the FHR-1:fH ratio were elevated in IgAN and were associated with progressive disease

While fH is the major negative regulator of C3 activation via the complement alternative pathway, FHR-1 is postulated to act as a positive regulator by antagonizing the effect of fH, a process termed fH deregulation.^17^, ^18^, ^19^, ^24^ To investigate fH deregulation in IgAN, we measured both plasma fH and FHR-1 levels. The median plasma FHR-1 level was increased in IgAN patients compared with that in healthy controls, whereas the plasma fH level did not differ (Table 1). Notably, the relative abundance of these proteins differed between patients and healthy controls, and the FHR-1:fH ratio was significantly increased in IgAN patients (Table 1).

**Figure 1:**
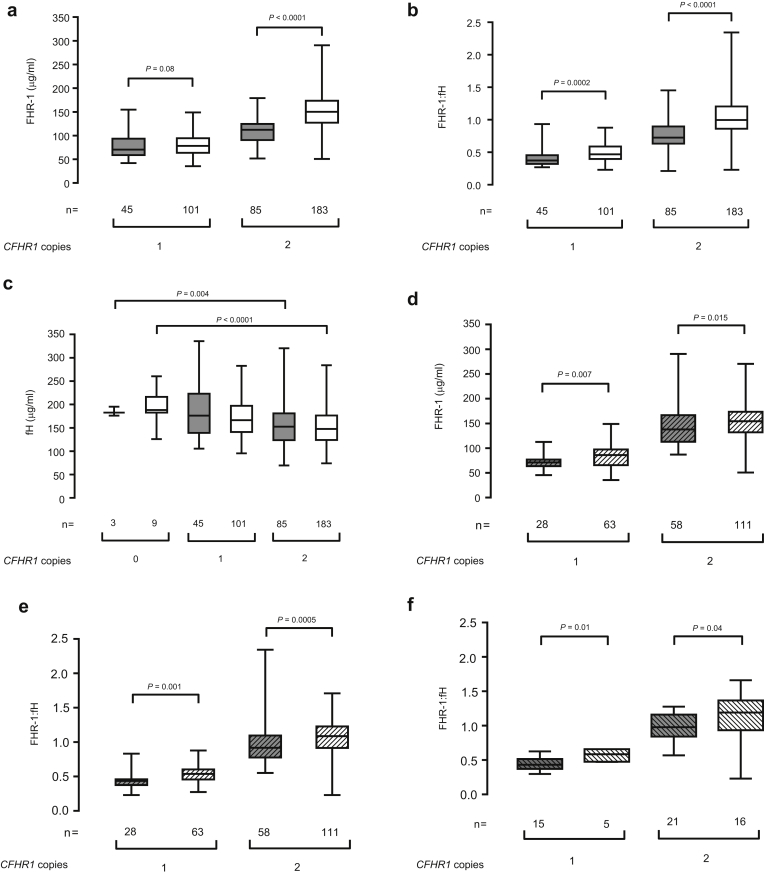
**The plasma factor H-related protein 1 (FHR-1) levels and the FHR-1:factor H (fH) ratio are elevated in IgA nephropathy (IgAN) and are associated with a progressive disease.** (**a**) The plasma FHR-1 levels, (**b**) FHR-1:fH ratio, and (**c**) fH levels in healthy controls (gray boxes) and IgAN patients (white boxes) stratified according to the *CFHR1* gene copy number. Plasma FHR-1 was undetectable in patients with 0 *CFHR1* gene copy number. The plasma FHR-1 levels and FHR-1:fH ratio were significantly different (*P* < 0.001) between individuals with either 1 or 2 *CFHR1* gene copy numbers in both the control and IgAN cohorts. (**d**) Comparison of plasma FHR-1 levels and (**e**) FHR-1:fH ratio between patients with stable (dashed gray boxes) and those with progressive (dashed white boxes) IgAN stratified according to the *CFHR1* gene copy number. (**f**) Comparison of the FHR-1:fH ratio between patients with stable (dashed gray boxes) and those with progressive (dashed white boxes) IgAN after immunosuppression therapy. The bar represents the median value, box represents the interquartile range, and whiskers represent the range of values. *P* values derived using Mann-Whitney test.

Because the presence of the del*CFHR3-1* allele will influence circulating FHR-1 levels (most clearly the absence of the protein in del*CFHR3-1* homozygotes), we stratified patients according to the *CFHR1* gene copy number. Patients with 2 copies of the *CFHR1* gene had higher FHR-1 levels compared with genotype-matched healthy controls (Figure 1a). This difference was not observed in patients with 1 *CFHR1* gene copy number (Figure 1a). However, the FHR-1:fH ratio was significantly higher in patients than in healthy controls, irrespective of the *CFHR1* gene copy number (Figure 1b). FHR-1 was undetectable in deletion homozygotes (n = 12, including 9 patients). Plasma fH levels remained similar between patients and controls when stratified according to the *CFHR1* gene copy number (Figure 1c). As previously reported,^15^, ^25^ fH levels were higher in del*CFHR3-1* homozygotes (Figure 1c).

We next examined if FHR-1 levels and the FHR-1:fH ratio differed between patients with stable IgAN and those with progressive IgAN. Irrespective of the *CFHR1* gene copy number, compared with patients with stable IgAN, those with progressive IgAN had significantly elevated plasma FHR-1 levels (Figure 1d) and FHR-1:fH ratios (Figure 1e), whereas fH levels did not differ (Table 1). In addition, the FHR-1:fH ratio was elevated in patients who had progressive compared with stable disease following immunosuppression treatment (Figure 1f).

### Plasma FHR-1 was negatively correlated with eGFR but remained elevated in IgAN patients with normal eGFR

**Figure 2:**
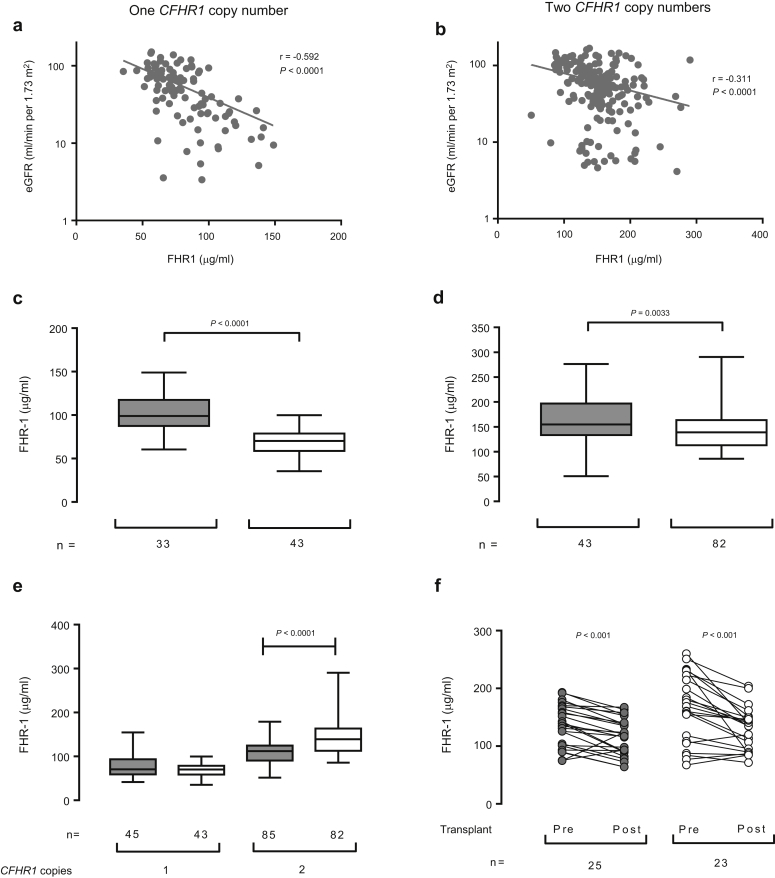
**Factor H-related protein 1 (FHR-1) is negatively correlated with the estimated glomerular filtration rate (eGFR) but remains elevated in IgA nephropathy (IgAN) patients with normal eGFR.** Correlation between plasma FHR-1 levels and eGFR after logarithmic transformation in IgAN patients with either 1 (**a**) or 2 (**b**) *CFHR1* gene copy numbers. *P* values derived from Spearman’s rank correlation. Plasma FHR-1 levels in IgAN patients with eGFR of <30 (gray boxes) or >60 (white boxes) ml/min per 1.73 m^2^ stratified according to 1 (**c**) or 2 (**d**) *CFHR1* gene copy number. (**e**) Comparison of the plasma FHR-1 levels between healthy controls (gray boxes) and IgAN patients with eGFR of >60 ml/min per 1.73 m^2^ (white boxes) stratified according to the *CFHR1* gene copy number. The bar represents the median value, box represents the interquartile range, and whiskers represent the range of values. *P* values derived from Mann-Whitney test. (**f**) Paired FHR-1 levels before (Pre) and after (Post) renal transplantation in a cohort of patients with autosomal dominant polycystic kidney disease (ADPKD) (gray circles, n = 25) and IgAN (white circles, n = 23). All patients were homozygous for the major allele rs6677604, consistent with 2 *CFHR1* gene copy numbers.

Given that we observed higher FHR-1 levels in patients with progressive IgAN, we next determined if FHR-1 levels were influenced by renal impairment. We stratified patients by the *CFHR1* gene copy number and assessed the correlation between FHR-1 levels and eGFR (Figure 2). We detected a negative correlation between plasma FHR-1 levels and eGFR in patients with either 1 (Figure 2a) or 2 (Figure 2b) *CFHR1* gene copy number. When we stratified patients into those with eGFR of <30 ml/min per 1.73 m^2^ and those with eGFR of >60 ml/min per 1.73 m^2^, FHR-1 levels were significantly higher in those with eGFR of <30 ml/min per 1.73 m^2^ (Figure 2c and d, 1 and 2 *CFHR1* copy numbers, respectively). To determine if FHR-1 levels were higher in IgAN patients before the development of renal impairment, we compared FHR-1 levels between IgAN patients with normal eGFR and healthy controls (Figure 2e). Higher FHR-1 levels were observed in patients with 2 *CFHR1* gene copy numbers and normal eGFR than in genotype-matched healthy controls (Figure 2e). This difference was not observed in patients with 1 *CFHR1* gene copy number (Figure 2e). We next assessed FHR-1 levels before and after renal transplantation in patients with either biopsy-proven IgAN or autosomal dominant polycystic kidney disease (ADPKD). The cohorts had comparable pretransplant characteristics (Supplementary Table S1), and no patients had a clinical diagnosis of delayed graft function, transplant rejection, or disease recurrence at the sampling time. Both groups showed significant reduction in serum FHR-1 levels after renal transplantation (Figure 2f). Altogether, our data indicate that both the diagnosis of IgAN and eGFR are independently associated with higher FHR-1 levels. Notably, there was no significant correlation between eGFR and plasma fH levels in our IgAN cohort (Supplementary Figure S1C).

### Serum FHR-5 was slightly elevated in IgAN but was not correlated with eGFR

**Figure 3:**
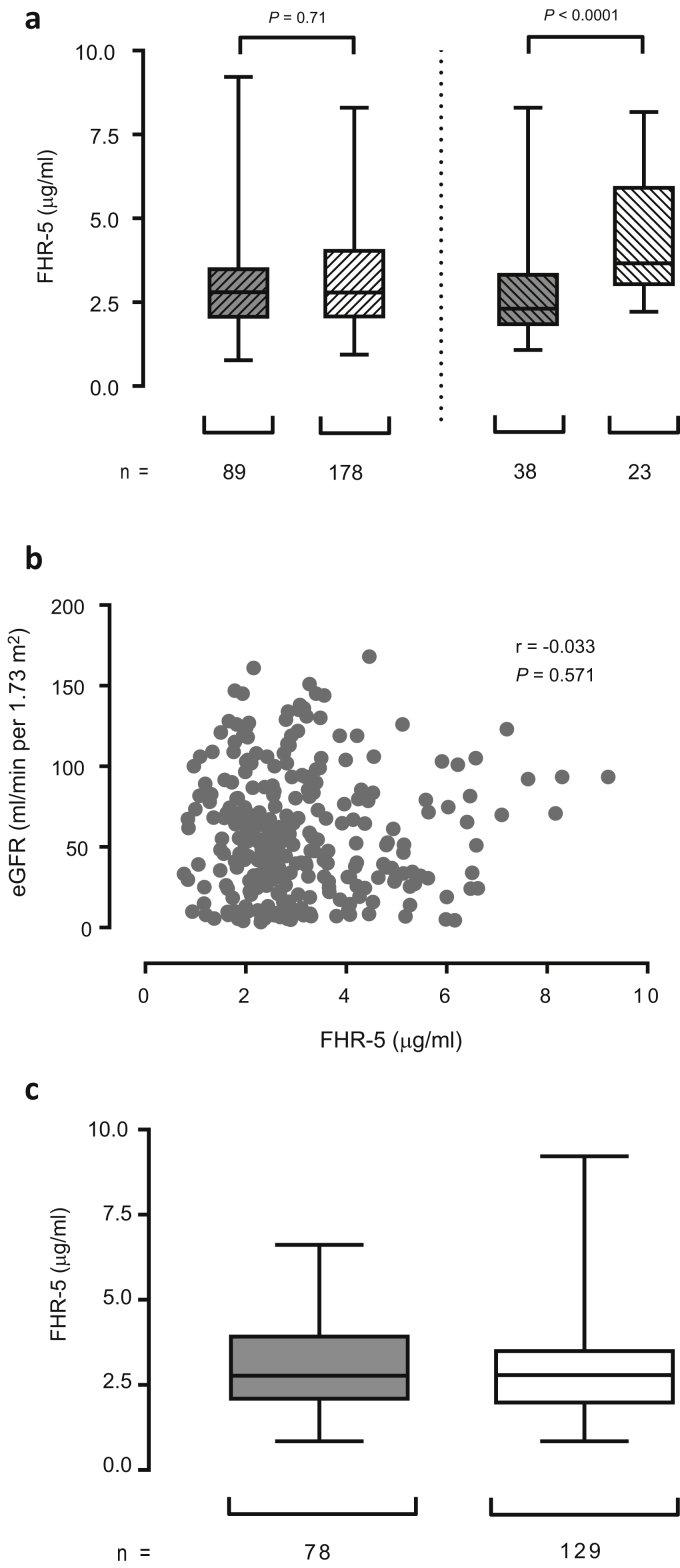
**Serum factor H-related protein 5 (FHR-5) levels are associated with IgA nephropathy (IgAN) severity following immunosuppression therapy and are not correlated with estimated glomerular filtration rate (eGFR).** (**a**) Left panel, Comparison of serum FHR-5 levels between stable (dashed gray boxes) and progressive (dashed white boxes) IgAN. Right panel, Comparison of serum FHR-5 between patients with stable (dashed gray boxes) and those with progressive (dashed white boxes) IgAN after immunosuppression therapy. (**b**) Correlation between serum FHR-5 levels and eGFR in IgAN patients. (**c**) Serum FHR-5 levels in IgAN patients with eGFR of <30 (gray boxes) or >60 (white boxes) ml/min per 1.73 m^2^. The bar represents the median value, box represents the interquartile range, and whiskers represent the range of values. *P* values derived using Mann-Whitney test.

Abnormalities in FHR-5 have been shown to be associated with C3 glomerulopathy (C3G), a complement-mediated kidney disease with phenotypic similarities to IgAN.^26^, ^27^ In addition, FHR-5 is associated with fH deregulation *in vitro*.^19^ We measured serum FHR-5 levels in our IgAN cohort. The median FHR-5 level was higher in IgAN patients than in healthy controls (Table 1), but the magnitude of the difference was small. The median FHR-5 levels were 2.46 and 2.74 μg/ml in the healthy controls and IgAN patients, respectively (difference between medians, 0.28 μg/ml, 95% confidence interval, 0.01–0.48 μg/ml). Furthermore, we did not detect any difference in FHR-5 levels between stable and progressive IgAN patients (Figure 3a and Table 1). However, patients with progressive disease following immunosuppression treatment had significantly higher FHR-5 levels than patients who improved to meet stable IgAN criteria after immunosuppression (Figure 3a). Unlike FHR-1, we did not find an association between serum FHR-5 levels and eGFR (Figure 3b and c). Moreover, we found no significant difference in the FHR-5:fH ratio between patients and healthy controls or between progressive and stable IgAN (Supplementary Figure S2).

### Serum FHR-5 levels correlated with histologic markers of renal injury

**Figure 4:**
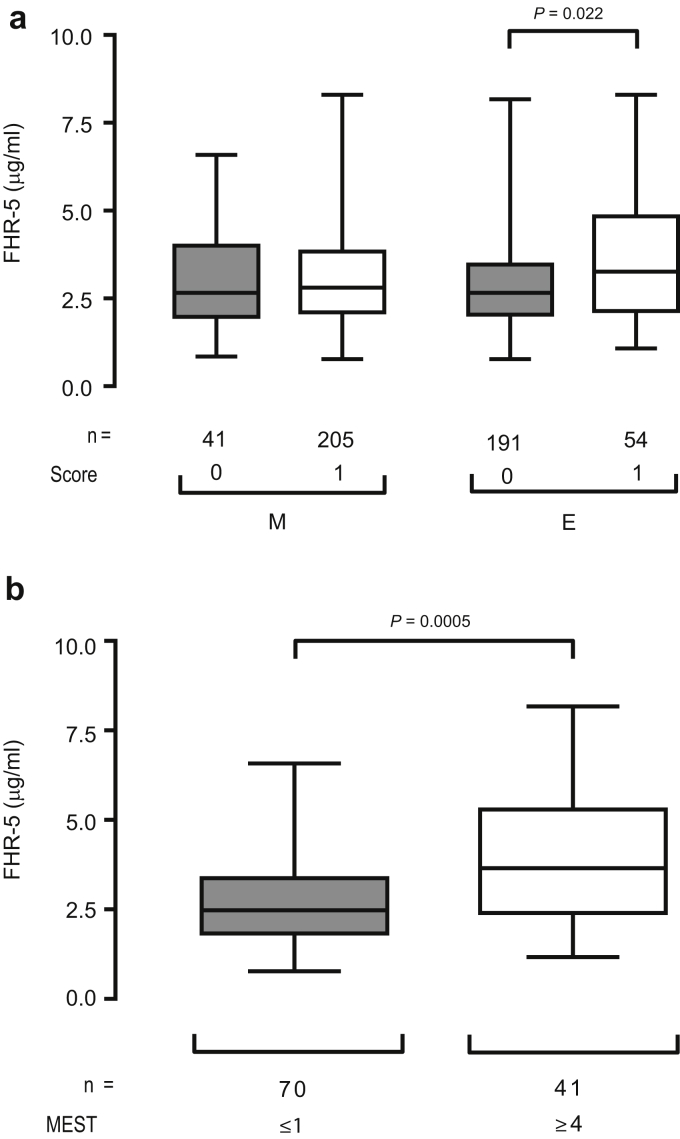
**Serum factor H-related protein 5 (FHR-5) levels are correlated with histologic markers of disease severity in IgA nephropathy (IgAN).** (**a**) Left panel, Serum FHR-5 levels in IgAN patients without (M0, gray box) and with (M1, white box) renal biopsy evidence of mesangial hypercellularity (denoted M). Right panel, Serum FHR-5 levels in IgAN patients without (E0, gray box) and with (E1, white box) biopsy evidence of endocapillary hypercellularity (denoted E). (**b**) Serum FHR-5 levels in IgAN patients with diagnostic renal biopsy MEST scores of 1 or less (gray box) or at least 4 (white box). The bar represents the median value, box represents the interquartile range, and whiskers represent the range of values. *P* values derived using Mann-Whitney test.

To explore the significance of the changes in serum FHR-5 levels, we assessed the correlation between FHR-5 levels and validated markers of histologic injury in IgAN, according to the Oxford classification.^28^ Serum FHR-5 levels at recruitment were significantly higher in patients with endocapillary hypercellularity score E1 at diagnosis than in those with no endocapillary hypercellularity (E0) (Figure 4a, right panel). Serum FHR-5 levels in IgAN patients with (M1) and without (M0) renal biopsy evidence of mesangial hypercellularity did not differ (Figure 4a, left panel). The total MEST score is calculated by adding the scores for mesangial hypercellularity (M), endocapillary hypercellularity (E), segmental sclerosis (S), and tubular atrophy (T). Serum FHR-5 levels were higher in IgAN patients with MEST score of 4 than in those with MEST score of 1 (Figure 4b). Unlike FHR-5, our data demonstrated that FHR-1 levels were influenced by eGFR. Therefore, we did not consider it valid to assess associations between histologic markers and plasma FHR-1 levels because eGFR would differ between the diagnostic renal biopsy and study plasma in many patients.

## Discussion

The mechanism underlying the robust association between the del*CFHR3-1* allele and reduced susceptibility to IgAN is unclear. While the role of fH as a critical negative regulator of C3 activation through the complement alternative pathway is well established, the biological roles of the FHR proteins have, until recently, been poorly understood.^17^, ^18^, ^19^ In fact, the common occurrence of the complete absence of FHR-1 and FHR-3 in healthy individuals with the del*CFHR3-1* allele in homozygosity has suggested that these 2 FHR proteins are biologically redundant. Insights into key roles of FHR-1 and FHR-3 in diseases have been derived from the association between del*CFHR3-1* alleles and protection from not only IgAN but also age-related macular degeneration.^25^ Furthermore, abnormal FHR proteins are associated with familial cases of C3G, a condition wherein complement-mediated renal injury is derived from abnormal regulation of alternative pathway activation.^20^, ^26^, ^29^, ^30^, ^31^ *In vitro* data have shown that FHR-1^17^ and FHR-5^19^ compete with fH for binding to activated C3 (termed C3b). Unlike fH, interaction of C3b with either FHR-1 or FHR-5 allows continued complement activation, preventing the inhibitory actions of fH, a phenomenon referred to as fH deregulation. We considered that the association between del*CFHR3-1* alleles and IgAN can be explained by fH deregulation. We hypothesized that reduced (or absent) FHR-1 levels result in a reduction or absence of fH deregulation and consequently less complement-mediated renal injury.

In this study, we first explored the association between FHR-1 and fH levels in patients with either stable or progressive IgAN. We found that FHR-1 levels and importantly, the FHR-1:fH ratio, were higher in IgAN patients than in healthy controls. These data are replicated in a separate IgAN cohort (Tortajada A, Gutierrez E, Goicoechea de Jorge E, et al. Elevated factor H-related 1 and occurrence of factor H pathogenic variants in IgA nephropathy. *Kidney International,* submitted for publication). In addition, irrespective of the *CFHR1* gene copy number, patients with progressive IgAN had significantly elevated plasma FHR1/fH ratios compared to patients with stable IgAN. These data are consistent with our hypothesis that reduced fH deregulation is associated with favorable outcomes in IgAN. We categorized the patient cohort into those with progressive IgAN and those with stable IgAN. Our criteria were designed to enable us to identify patients with immunologically active disease. The criteria included eGFR loss without additional renal pathology and histologic features of glomerular inflammation, such as the presence of endocapillary hypercellularity and cellular crescents. Although this inevitably excluded a subset of patients (those we could not reliably categorize as either stable or progressive at study entry), our robust classification enabled us to detect differences in FHR-1 levels and the FHR-1:fH ratio. Although changes in these parameters correlated with worse disease outcomes, the levels showed large overlap between the groups, precluding their use for patient stratification. In addition, our cohort included 9 individuals (3 with stable IgAN, 4 with progressive IgAN, and 2 who did not fulfil the criteria) with del*CFHR3-1* allele homozygosity, demonstrating that even in the complete absence of FHR-1 and FHR-3, renal injury that requires renal biopsy and hospital follow-up can occur. This is consistent with previous studies^16^ and indicates that factors independent of fH deregulation can drive the disease.

We did not measure FHR-3 levels because of the lack of a reliable assay. FHR-3 is not clearly associated with fH deregulation, and its role remains unclear; it can interact with the meningococcus fH-binding protein and through competition with fH, influence the complement-mediated clearance of meningococcal strains and hence meningococcal disease severity.^32^ Through interaction with its ligand C3d, FHR-3 may influence B-cell regulation through a B-cell receptor complex (CD19/CD21/CD81), but the association with IgAN is unclear.^33^ Notably, despite the interaction with C3d, this effect was not observed for FHR-1.

We found a negative correlation between eGFR and plasma FHR-1 levels measured at study recruitment. When patients with normal eGFR were compared with genotype-matched healthy controls, patients with 2 *CFHR1* gene copy numbers and normal eGFR had higher FHR-1 levels. Both IgAN patients and ADPKD patients showed reduced FHR-1 levels coincidental with increased eGFRs after renal transplantation. This indicated that the diagnosis of IgAN and eGFR are independently associated with higher FHR-1 levels. Moreover, this conclusion is supported by the findings in a study that assessed FHR-1 levels in IgAN and polycystic renal disease (Tortajada A, Gutierrez E, Goicoechea de Jorge E, et al. Elevated factor H-related 1 and occurrence of factor H pathogenic variants in IgA nephropathy. *Kidney International,* submitted for publication). Although we consider the association to be robust, we currently do not know why FHR-1 increases as eGFR decreases. Nevertheless, this would be predicted to further enhance fH deregulation and aggravate disease. It is predicted that this could be applicable to any glomerular pathology in which there is complement-mediated injury and that there are more general implications beyond IgAN. Because FHR-1 levels were influenced by eGFR and diagnostic renal biopsy was not coincident with the timing of the study blood sample in our cohort, we did not analyze the association between FHR-1 and histologic changes in the renal biopsy. A prospective study will be required to clarify the contribution of FHR-1 to histologic changes before decreases in eGFR.

As published data showed that FHR-5 can mediate fH deregulation^19^ and studies indicated the strong association between FHR-5 mutation and C3G,^26^, ^30^, ^31^ we measured serum FHR-5 levels and the FHR-5:fH ratio. Notably, there are strong phenotypic similarities between familial C3G associated with FHR-5 mutations and IgAN.^27^ Despite a significant increase in serum FHR-5 levels between IgAN patients and healthy controls, the magnitude of the difference was very small and of doubtful biological significance. In addition, we did not identify any difference in FHR-5 levels between patients with stable disease and those with progressive disease. However, when we analyzed only patients treated with immunosuppression therapy, FHR-5 levels were higher in those with ongoing progressive IgAN. The significance of this is unclear and requires confirmation. Unlike the FHR-1:fH ratio, we did not detect any correlation between the FHR-5:fH ratio and disease outcome. Because eGFR did not influence the serum FHR-5 levels, we assessed the correlations between serum FHR-5 levels and validated histologic markers of renal injury in IgAN. Serum FHR-5 levels were associated with endocapillary hypercellularity independent of mesangial hypercellularity, a histologic marker of active inflammation.^34^ They were also associated with higher overall Oxford classification of IgAN MEST scores.^35^ To further understand these associations, it would be necessary to analyze the pattern and degree of FHR-5 deposition in renal tissues. In this respect, it is notable that FHR-5 was detected in association with complement C3 in patients with IgAN. Notably, the association between FHR-5 and C3 was also observed in membranous nephropathy, lupus nephritis, and postinfectious nephritis, indicating that this phenomenon is not specific to IgAN.^36^

The limitations of our dataset include the difference in timing between diagnostic renal biopsy and study sample collection and the lack of serial blood samples from patients. Additionally, it would be important to investigate the relative amounts of C3, fH, FHR-1, and FHR-5 in renal tissues in IgAN.

Current assessment of IgAN patients involves the use of nonspecific clinical and histologic markers to identify patients likely to improve with immunosuppression therapy. However, the benefit of the currently recommended 6-month corticosteroid treatment for persistently proteinuric, noncrescentic IgAN^37^ has not been conclusively demonstrated.^38^ Our data, together with those of a separate cohort (Tortajada A, Gutierrez E, Goicoechea de Jorge E, et al. Elevated factor H-related 1 and occurrence of factor H pathogenic variants in IgA nephropathy. *Kidney International,* submitted for publication), associate circulating FHR-1 levels and the FHR-1:fH ratio with IgAN severity. Our findings need corroboration with histologic data but nevertheless suggest that fH deregulation contributes to IgAN progression and support further investigation into this hypothesis. If it can be shown that complement-mediated kidney injury in IgAN is influenced by FHR-1 and mechanistically understood, the modulation of FHR-1 might be therapeutic in progressive IgAN.

## Materials and Methods

### Study cohort and clinical measurements

The Causes and Predictors of Outcome in IgA Nephropathy study is a retrospective cohort UK study of patients with biopsy-proven IgAN ethically approved by the UK National Research Ethics Service Committee (14/LO/0155). The inclusion criteria were availability of the renal biopsy report and relevant clinical data. In this study, 334 patients were recruited, and after excluding 40 patients, 294 were analyzed. The reasons for exclusion were alternative diagnosis (n = 4), no biopsy immunostaining completed (n = 5), no biopsy report available (n = 3), and no creatinine measurement at enrollment (n = 28).

Progressive disease was considered if at least one of the following criteria was present: (i) progression to end-stage renal disease without histologic evidence of a second pathology causing renal impairment, (ii) renal biopsy evidence of endocapillary hypercellularity, (iii) renal biopsy evidence of cellular and/or fibrocellular crescents; (iv) treatment with immunosuppressants (including corticosteroids) for native IgAN, (v) clinical Henoch-Schonlein purpura, unless spontaneous resolution and >20 years of follow-up with “stable” criteria, (vi) 50% loss of eGFR or an average annual eGFR loss of >5 ml/min per 1.73 m^2^ without evidence of a second pathology causing renal impairment. Stable disease was considered if none of the criteria for progressive IgAN were met and if all of the following criteria were met: (i) urine protein-to-creatinine ratio of <100 units or daily proteinuria of <1 g/24 h, (ii) combined Oxford classification MEST score of <3, and (iii) an average annual eGFR loss of <3 ml/min per 1.73 m^2^.

All patients in the transplantation cohorts received a renal transplant and underwent clinical follow-up at Imperial College Healthcare NHS Trust. They received posttransplant immunosuppression therapy and clinical care as per local guidelines. All ADPKD patients had a radiological diagnosis. All transplant patients provided consent for the storage of serum and plasma samples that were surplus to clinical diagnostic requirements and their subsequent use for research. Blood samples were obtained within 4 weeks before and between 12 and 16 weeks after transplantation. Two transplant ADPKD patients, both of whom had the AA rs6677604 single-nucleotide polymorphism genotype, had undetectable FHR-1 levels before and after transplantation.

Healthy control samples were obtained from healthy volunteer donors and from members of the TwinsUK cohort,^39^ a cohort of twins of Caucasian ethnicity. We randomly selected samples from only 1 individual of each pair of twins included in the cohort.

eGFR was calculated using the Chronic Kidney Disease Epidemiology Collaboration Creatinine Equation.^40^

### Assessment of *CFHR3* and *CFHR1* gene copy number

DNA was extracted from whole blood samples using QIAamp DNA Blood Mini Kits (Qiagen, Hilden, Germany). Quantitative real-time polymerase chain reaction (PCR) was performed using the ViiA Real-Time PCR System (Applied Biosystems, Foster City, CA). Copy number variation within the *CFHR3* and *CFHR1* genes was assessed using the Taqman Copy Number Real-Time Detection System (Applied Biosystems). Copy number variation calls were determined using the Copy Caller Software (Applied Biosystems). Assay readings were normalized to control samples, and the values were presented as mean ± SD. All probes were validated using genomic DNA from healthy controls with either heterozygous or homozygous polymorphic deletion of the *CFHR1* and *CFHR3* genes. The *CFHR1* gene copy number in the renal transplant cohort was inferred from the rs6677604 genotype, which is in linkage disequilibrium with the *CFHR1* and *CFHR3* gene copy number.^16^ Genotyping was performed using the Taqman genotyping assays (Applied Biosystems).

### Measurement of serum IgA and gd-IgA levels

Serum IgA levels were measured using enzyme-linked immunosorbent assay (ELISA) as previously described.^41^ The capture antibody was the F(ab’)2 fragment goat antihuman IgA (Jackson ImmunoResearch, West Grove, PA), and the detection antibody was the F(ab’)_2_ fragment biotinylated goat antihuman IgA1 (Jackson ImmunoResearch). Serum gd-IgA1 levels were measured using a lectin-based ELISA as previously described.^41^ The capture antibody was a polyclonal rabbit antihuman IgA (Dako, Glostrup, Denmark). The detection involved *Helix aspersa* agglutinin-biotin (Sigma, Darmstadt, Germany), followed by poly-streptavidin horseradish peroxidase (Pierce, Waltham, MA). The intraclass correlation coefficient for the IgA assay was 0.74 (95% confidence interval, 0.63–0.83), and that for the gd-IgA1 assay was 0.89 (95% confidence interval, 0.73–0.95).

### Measurement of plasma fH and FHR-1 levels and serum FHR-5 levels

We used a sandwich ELISA designed by Tortajada *et al.* (Tortajada A, Gutierrez E, Goicoechea de Jorge E, et al. Elevated factor H-related 1 and occurrence of factor H pathogenic variants in IgA nephropathy. *Kidney International,* submitted for publication) to measure the plasma fH and FHR-1 levels. Although FHR-1 and FHR-2 can form homodimers, heterodimers, and heterooligomeric molecules *in vivo*, the levels detected by this ELISA are referred to as FHR-1 levels because FHR-1 is the major component in these complexes. The capture antibody was a rabbit polyclonal antibody that recognizes both fH and FHR-1. fH was detected with a mouse monoclonal anti-fH antibody that recognizes SCR10 and SCR11 of fH. FHR-1 was detected using a mouse monoclonal antibody that recognizes an epitope within SCR1 and SCR2 of FHR-1 (both provided by Professor Santiago Rodriguez de Cordoba, Madrid). The interassay coefficient of variation was 8.7 for fH ELISA and 9.9 for FHR-1 ELISA.

FHR-5 levels were measured using ELISA. The capture antibody was a rabbit monoclonal anti-FHR-5 antibody (Abcam, Cambridge, UK). FHR-5 was detected using a mouse monoclonal anti-FHR-5 antibody (Abcam). The interassay coefficient of variation was 12.1.

### Statistical analysis

Normally distributed continuous variables were tested using unpaired *t*-test, and pre- and posttransplant levels were compared using paired *t*-test. Continuous variables with skewed distribution were tested using Mann-Whitney *U* test, and confidence intervals for differences between medians were calculated using the Hodges-Lehmann method. Categorical data was tested using chi-square test and Fisher’s exact test (samples, <10). We applied Pearson’s correlations and simple linear regression to log eGFR and FHR-1, and Spearman’s rank correlation to eGFR and gd-IgA1. A *P* value <0.05 was considered to be statistically significant. We used GraphPad Prism version 6.00 for Windows (GraphPad Software, La Jolla, CA) for all analyses.

## Disclosure

All the authors declare no competing interests.

## Collaborator List and Affiliations

Dr. A. Ahmed, Royal Preston Hospital, Lancashire, UK.

Dr. L. Baines, Freeman Hospital, Newcastle upon Tyne, UK.

Dr. C. Bingham, Royal Devon and Exeter Hospital, Exeter, UK.

Prof S. Bhandari, Hull Royal Infirmary, Hull, UK.

Dr. A. Bow, Cumberland Infirmary, Carlisle, UK.

Dr. A. Chan, Broomfield Hospital, Chelmsford, Essex, UK.

Dr. G. Cserep, Colchester General Hospital, Colchester, Essex, UK.

Dr. D. de Takats, Royal Stoke University Hospital, Stoke-on-Trent, UK.

Dr. S. Dickinson, Royal Cornwall Hospital, Truro, Cornwall, UK.

Dr. C. Goldsmith, Aintree University Hospital, Liverpool, UK.

Dr. S. Lawman, Royal Sussex County Hospital, Brighton, UK.

Dr. S. Mitra, Manchester Royal Infirmary, Manchester, UK.

Dr. R. Montero, Guys Hospital, London, UK.

Dr. A. Mooney, St James’s University Hospital, Leeds, UK.

Dr. A. Power, Southmead Hospital, Bristol, UK.

Dr. C. Wroe, James Cook University Hospital, Middlesbrough, UK.

## Supplementary Material

Figure S1 (**A**) Serum IgA (left panel) and galactose-deficient IgA1 (gd-IgA1; right panel) levels in patients with stable (gray dashed box) and progressive (white dashed box) IgA nephropathy (IgAN) after immunosuppression therapy. The progressive IgAN cohort shows higher median serum gd-IgA1 levels. (**B**) Correlation of estimated glomerular filtration rate (eGFR) and serum gd-IgA1 levels in IgAN patients (n = 293). A negative correlation was calculated with Spearman’s rank correlation, and the black line represents the correlation equation. (**C**) Comparison of serum eGFR and plasma factor H (fH) at the same sampling point in IgAN patients (n = 294) shows a small and insignificant correlation between eGFR and plasma fH levels.

Figure S2 The plasma factor H-related protein 5 (FHR-5):factor H (fH) ratio is not associated with IgA nephropathy (IgAN). (**A**) The FHR-5:fH ratio in healthy controls and IgAN patients. We did not identify a significant difference in plasma FHR-5:fH ratio between healthy controls (gray box) and IgAN patients (white box). (**B**) The FHR-5:fH ratio in stable and progressive IgAN. There was no significant difference in the plasma FHR-5:fH ratio between patients with stable (gray boxes) and those with progressive (white boxes) IgAN for the same *CFHR1* genotype.

Table S1 Cohort characteristics for patients with IgA nephropathy (IgAN) or autosomal dominant polycystic kidney disease (ADPKD) who received a renal transplant. Values within parentheses represent interquartile range. ESRD, end-stage renal disease. ^a^*P* < 0.001 compared with the pretransplant levels. *rs6677604 tags the *CFHR3-1* deletion.
