# Supplementary material for: Circulating complement factor H–related proteins 1 and 5 correlate with disease activity in IgA nephropathy
Source: Kidney Int. 2017 Oct;92(4):942–52. doi: 10.1016/j.kint.2017.03.043 (PMC5611987; doi:10.1016/j.kint.2017.03.043)
Supplement: Figure S1 — (A) Serum IgA (left panel) and galactose-deficient IgA1 (gd-IgA1; right panel) levels in patients with stable (gray dashed box) and progressive (white dashed box) IgA nephropathy (IgAN) after immunosuppression therapy. The progressive IgAN cohort shows higher median serum gd-IgA1 levels. (B) Correlation of estimated glomerular filtration rate (eGFR) and serum gd-IgA1 levels in IgAN patients (n = 293). A negative correlation was calculated with Spearman’s rank correlation, and the black line represents the correlation equation. (C) Comparison of serum eGFR and plasma factor H (fH) at the same sampling point in IgAN patients (n = 294) shows a small and insignificant correlation between eGFR and plasma fH levels. [file mmc1.pptx]

## Slide 1
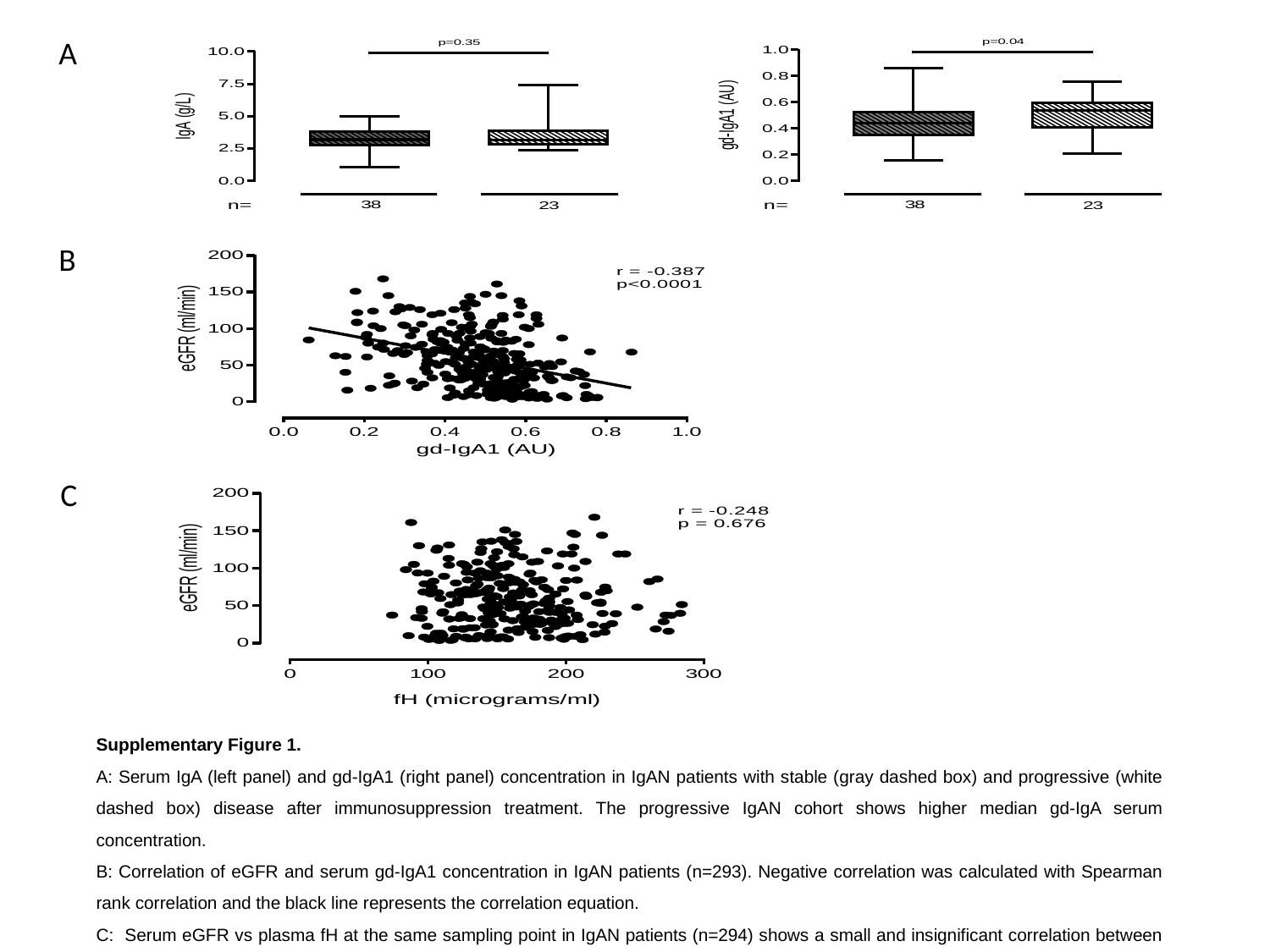

A
B
C
Supplementary Figure 1.
A: Serum IgA (left panel) and gd-IgA1 (right panel) concentration in IgAN patients with stable (gray dashed box) and progressive (white dashed box) disease after immunosuppression treatment. The progressive IgAN cohort shows higher median gd-IgA serum concentration.
B: Correlation of eGFR and serum gd-IgA1 concentration in IgAN patients (n=293). Negative correlation was calculated with Spearman rank correlation and the black line represents the correlation equation.
C: Serum eGFR vs plasma fH at the same sampling point in IgAN patients (n=294) shows a small and insignificant correlation between eGFR and plasma fH concentration
