# Supplementary material for: Circulating complement factor H–related proteins 1 and 5 correlate with disease activity in IgA nephropathy
Source: Kidney Int. 2017 Oct;92(4):942–52. doi: 10.1016/j.kint.2017.03.043 (PMC5611987; doi:10.1016/j.kint.2017.03.043)
Supplement: Figure S2 — The plasma factor H-related protein 5 (FHR-5):factor H (fH) ratio is not associated with IgA nephropathy (IgAN). (A) The FHR-5:fH ratio in healthy controls and IgAN patients. We did not identify a significant difference in plasma FHR-5:fH ratio between healthy controls (gray box) and IgAN patients (white box). (B) The FHR-5:fH ratio in stable and progressive IgAN. There was no significant difference in the plasma FHR-5:fH ratio between patients with stable (gray boxes) and those with progressive (white boxes) IgAN for the same CFHR1 genotype. [file mmc2.pptx]

## Slide 1
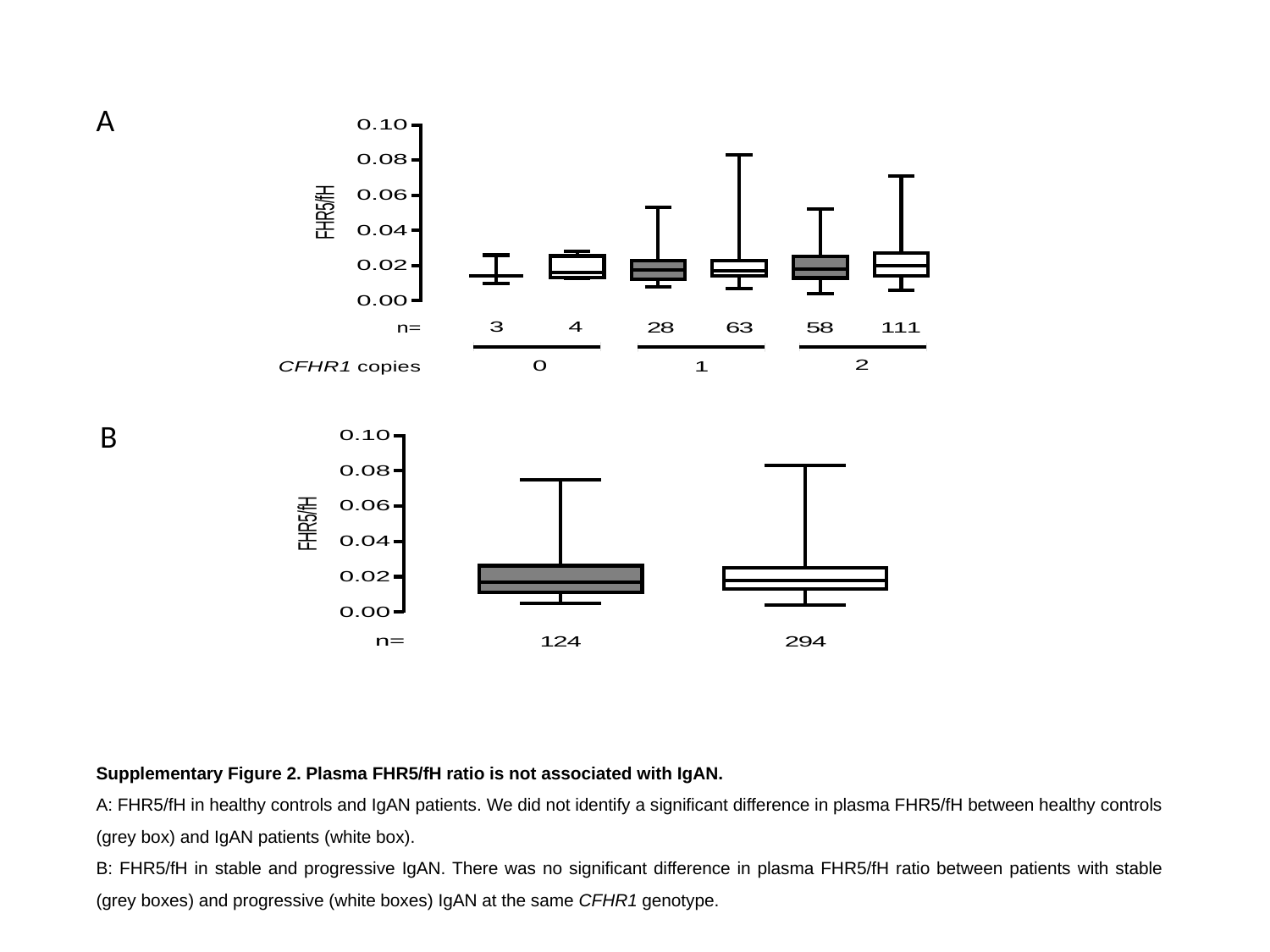

A
B
Supplementary Figure 2. Plasma FHR5/fH ratio is not associated with IgAN.
A: FHR5/fH in healthy controls and IgAN patients. We did not identify a significant difference in plasma FHR5/fH between healthy controls (grey box) and IgAN patients (white box).
B: FHR5/fH in stable and progressive IgAN. There was no significant difference in plasma FHR5/fH ratio between patients with stable (grey boxes) and progressive (white boxes) IgAN at the same CFHR1 genotype.
