# Supplementary material for: Circulating complement factor H–related proteins 1 and 5 correlate with disease activity in IgA nephropathy
Source: Kidney Int. 2017 Oct;92(4):942–52. doi: 10.1016/j.kint.2017.03.043 (PMC5611987; doi:10.1016/j.kint.2017.03.043)
Supplement: Table S1 — Cohort characteristics for patients with IgA nephropathy (IgAN) or autosomal dominant polycystic kidney disease (ADPKD) who received a renal transplant. Values within parentheses represent interquartile range. ESRD, end-stage renal disease. aP < 0.001 compared with the pretransplant levels. *rs6677604 tags the CFHR3-1 deletion. [file mmc3.pptx]

## Slide 1
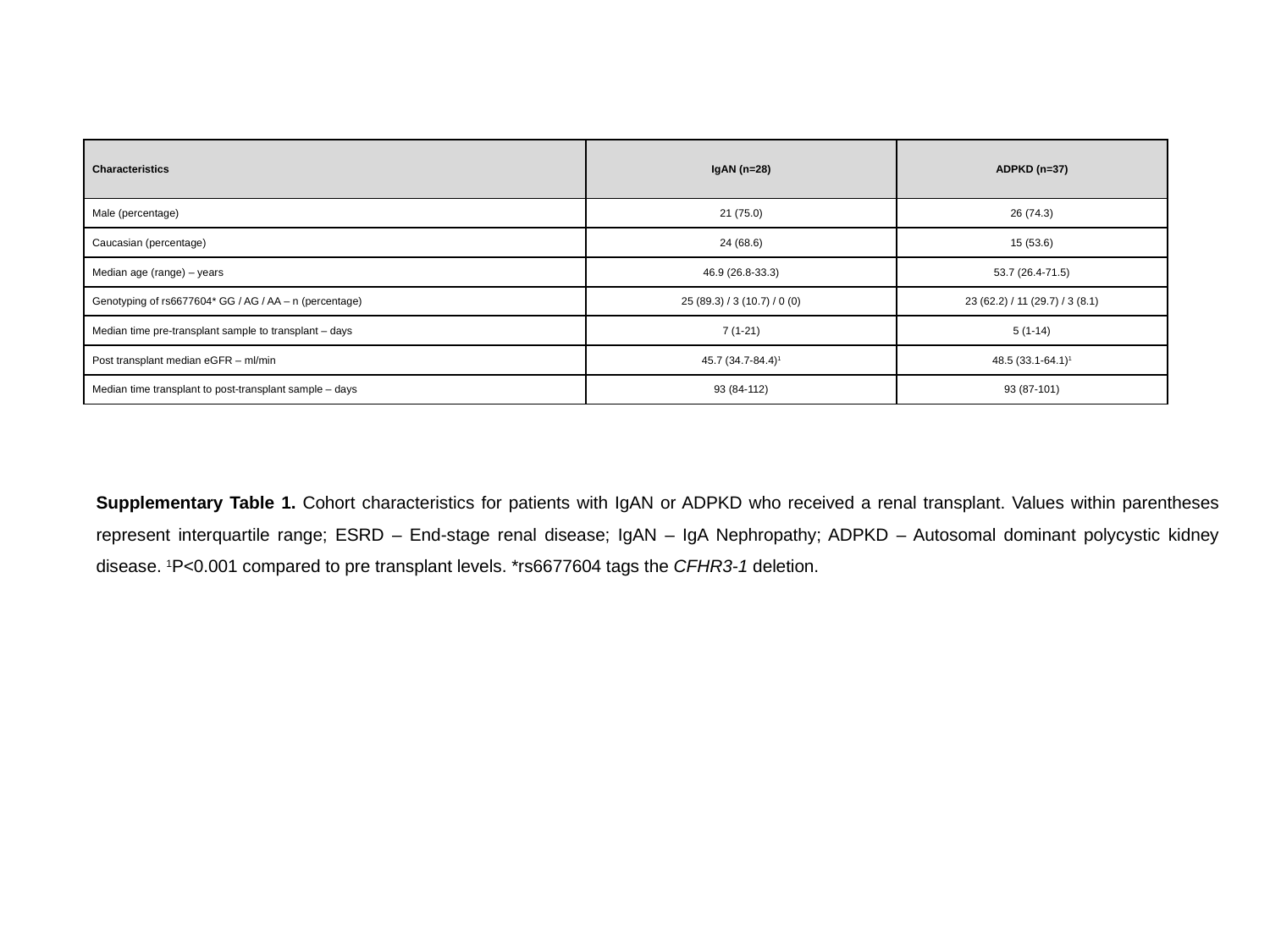

| Characteristics | IgAN (n=28) | ADPKD (n=37) |
| --- | --- | --- |
| Male (percentage) | 21 (75.0) | 26 (74.3) |
| Caucasian (percentage) | 24 (68.6) | 15 (53.6) |
| Median age (range) – years | 46.9 (26.8-33.3) | 53.7 (26.4-71.5) |
| Genotyping of rs6677604\* GG / AG / AA – n (percentage) | 25 (89.3) / 3 (10.7) / 0 (0) | 23 (62.2) / 11 (29.7) / 3 (8.1) |
| Median time pre-transplant sample to transplant – days | 7 (1-21) | 5 (1-14) |
| Post transplant median eGFR – ml/min | 45.7 (34.7-84.4)1 | 48.5 (33.1-64.1)1 |
| Median time transplant to post-transplant sample – days | 93 (84-112) | 93 (87-101) |
Supplementary Table 1. Cohort characteristics for patients with IgAN or ADPKD who received a renal transplant. Values within parentheses represent interquartile range; ESRD – End-stage renal disease; IgAN – IgA Nephropathy; ADPKD – Autosomal dominant polycystic kidney disease. 1P<0.001 compared to pre transplant levels. *rs6677604 tags the CFHR3-1 deletion.
